# Detection of Health-Related Events and Behaviours from Wearable Sensor Lifestyle Data Using Symbolic Intelligence: A Proof-of-Concept Application in the Care of Multiple Sclerosis

**DOI:** 10.3390/s21186230

**Published:** 2021-09-17

**Authors:** Thanos G. Stavropoulos, Georgios Meditskos, Ioulietta Lazarou, Lampros Mpaltadoros, Sotirios Papagiannopoulos, Magda Tsolaki, Ioannis Kompatsiaris

**Affiliations:** 1 Centre for Research & Technology Hellas, Information Technologies Institute, 6th Km Charilaou—Thermi, 57001 Thessaloniki, Greece; athstavr@iti.gr (T.G.S.); iouliettalaz@iti.gr (I.L.); lamprosmpalt@iti.gr (L.M.); ikom@iti.gr (I.K.); 2 School of Informatics, Aristotle University of Thessaloniki, 54124 Thessaloniki, Greece; 3 Department of Neurology III, Medical School, Aristotle University of Thessaloniki, 54124 Thessaloniki, Greece; spapagia@auth.gr; 4 Department of Neurology I, Medical School, Aristotle University of Thessaloniki, 54124 Thessaloniki, Greece; tsolakim1@gmail.com

**Keywords:** wearables, sensors, ontologies, symbolic reasoning, knowledge graphs, ehealth, multiple sclerosis

## Abstract

In this paper, we demonstrate the potential of a knowledge-driven framework to improve the efficiency and effectiveness of care through remote and intelligent assessment. More specifically, we present a rule-based approach to detect health related problems from wearable lifestyle sensor data that add clinical value to take informed decisions on follow-up and intervention. We use OWL 2 ontologies as the underlying knowledge representation formalism for modelling contextual information and high-level concepts and relations among them. The conceptual model of our framework is defined on top of existing modelling standards, such as SOSA and WADM, promoting the creation of interoperable knowledge graphs. On top of the symbolic knowledge graphs, we define a rule-based framework for infusing expert knowledge in the form of SHACL constraints and rules to recognise patterns, anomalies and situations of interest based on the predefined and stored rules and conditions. A dashboard visualizes both sensor data and detected events to facilitate clinical supervision and decision making. Preliminary results on the performance and scalability are presented, while a focus group of clinicians involved in an exploratory research study revealed their preferences and perspectives to shape future clinical research using the framework.

## 1. Introduction

Advances in sensing technology, namely the Internet of Things (IoT), combined with intelligent analysis and Artificial Intelligence (AI), promise to revolutionize care and mitigate the implications of chronic disease, often coupled with inaccessible or limited therapy options. A primary example and field of application is the ageing population support. According to the World Health Organization (WHO), people aged above 65 years old amount to 702.9 million in 2019—projected to reach 1548.9 million in 2050, where they would outnumber the children under the age of 14 [1]. This massive ageing population creates an impact on the social welfare and healthcare, causing a shift of the lifestyle and healthcare needs towards ailments associated with elders. The most prominent of those is dementia, which has great social and also financial impact [2]. People with dementia start to lose their ability to live independently as their condition progresses, which forces them to withdraw from their active role in society and the workforce, and eventually require daily assistance from informal carers like family members.

Although less prevalent in population, but similar to dementia, in terms of neurodegeneration and debilitation, is Multiple Sclerosis (MS). MS is an autoimmune disease affecting the brain and the spinal cord (central nervous system) disrupting communication with the rest of the body. As a result, the patient may suffer from symptoms such as vision impairment, muscle weakness, motor coordination and balance issues, cognitive deficit and mental health issues including depression. The effect on personal, social and professional life is detrimental. Its onset is not limited by age, but the majority lies between 15 and 45 years of age. Of two million cases worldwide, 65–70% are women [3]. While its course follows a relapse–remission cycle, 60–70% of the cases present secondary-progressive MS, a steady progression of symptoms regardless of remission periods.

Chronic disease, and particularly MS, require regular monitoring, effective support and personalized guidance to ensure the best possible outcomes [4], allowing specialised doctor’s to develop tailored treatment plans. Thus, the need for affordable, unobtrusive and easy-to-use healthcare solutions to support medical assessment and enable proactive intervention is growing [5]. In this quest, holistic and objective information to clinicians about patient health status can drive the development of personalised interventions to improve the patient’s health, psycho-social balance and quality of life, alleviating ailments and slowing down the progression of the disease. Solutions for effectively monitoring living conditions and habits, suggesting activities towards well-being and preventative actions, play a fundamental role in supporting individuals to maintain and continue their daily activities and live independently. Such solutions can also proactively help people who have no health problems to improve their lifestyle and adopt healthier living routines [6].

Wearable sensors are emerging as an effective tool for lifestyle monitoring and, thus, prevention, early detection and management of disease [7]. Coupled with the growing demand, they usher a paradigm shift toward digital self-management of chronic disease. Technological advancements grow the range of wearable sensors from smartwatches to textiles and smart glasses. Advancements in Artificial Intelligence (AI) and data analytics [8,9] allow detection and prediction of patterns and risk indicators over wearable sensor data and can enable more timely and efficient decision making. Still, interoperability and universal knowledge representation and management are needed to address the vast heterogeneity of data sources, devices and vendors to allow such knowledge extraction [10].

This paper describes a lightweight framework for detecting lifestyle and health-related problems, able to be easily configured and adapted to different environments, contexts, behaviours and monitoring objectives, supporting the integration of a variety of general purpose lifestyle wearable sensors. To achieve interoperability at different levels, we use OWL 2 ontologies [11] as the underlying knowledge representation formalism, generating interoperable Knowledge Graphs (KGs) that are aligned with existing vocabularies and conceptual models, such as the Sensor, Observation, Sample, and Actuator (SOSA) ontology [12], the Web Annotation Data Model (WADM) [13] and the Descriptions and Situations pattern [14]. The symbolic knowledge is further enriched with rules to derive logical consequences and semantically enrich the KGs. This involves the detection of behavioural patterns, habits and critical situations, encoding background knowledge and profile information using SHACL rules [15] to describe the patterns, resulting in a lightweight, standard-based reasoning and interpretation layer. Finally, intelligent and adaptive visualisations on a Clinician Dashboard further support the objective monitoring and evolution of the lifestyle behaviour.

The framework is applied in the context of the eHealth4MS project, presenting a use case for improving the care of Multiple Sclerosis (MS). The eHealth4MS project aims to develop an intelligent monitoring and decision support platform, integrating wearable sensors, data analytics and visualization dashboards. The platform will enable objective and reliable remote monitoring in non-clinical environments (e.g., patient homes), leading to more efficient, cost-effective and more accessible care, but also allowing self-management of their disease, increasing their quality of life. As such, we recruited patients with MS and clinician experts in the field to explore their views on the usefulness and relevance of the detected problems visualized on the Clinician Dashboard. Finally we evaluated performance in terms of scalability.

The contribution of our work can be summarised in the following:

- We integrate off-the-shelf wearable lifestyle sensors in a modular and extensible manner to extract data streams for steps (physical activity), sleep (onset, duration and stages) and heart rate.
- We reuse and combine different ontologies in a structural and modular manner to define the conceptual model of the underlying KGs at different levels. Capitalising on existing modelling standards (OWL 2 ontologies) and recommendations (SOSA, WADM, patterns), our framework supports the aggregated representation of observations, higher level knowledge and user-centred information to facilitate intelligent interlinking that can be easily shared and used inside rules.
- We use the latest W3C recommendation (SHACL) for encoding domain knowledge and monitoring patterns, defining the logic to monitor situations of interests.
- We present visualization on a web application dashboard for clinicians to review not only the original wearable data streams but also the detected health-related events in time, to enable decision making and tailored interventions for MS.
- We demonstrate use cases with real-world data from MS patients and perform an exploratory research study on a focus group of clinical experts to investigate their preferences and advice on how the framework fulfils their needs.
- We evaluate the performance in terms of scalability using real-world data.

The rest of the paper is structured as follows: Section 2 presents background and related work on eHealth solutions for MS and Semantic Web technologies. Section 3 gives an overview of the proposed framework, presenting the conceptual architecture and highlighting key concepts. Section 4 describes our approach for symbolic modelling and reasoning in order to detect situations of interest. Section 5 presents the dashboards that have been developed to assist clinical experts in decision making, while Section 6 presents evaluation results on the use of our framework on real-world data from MS patients. Finally, Section 7 concludes our work.

## 2. Background and Related Work

### 2.1. eHealth Solutions for MS

Several chronic diseases, such as dementia and cardiovascular disease, are lacking in pharmaceutical treatment or require lifestyle change. With spiralling costs and often lack of access to care, sensor and IoT technology is promising to provide timely, objective and remote monitoring, self-assessment and clinical decision support for intervention more efficiently and affordably. Several ehealth solutions exist to monitor and intervene in a single aspect of health, such as physical activity or exercise, sleep quality, serious games etc. [16]. Meanwhile, commercial solutions are also emerging in the market with lifestyle sensors available in retail and telehealth services in various mobile and web app stores. Despite that, analysis and extraction of features related to a pertaining disease that are clinically relevant are also lacking [17].

Particularly to MS, services are limited to telehealth, facilitating doctor and patient communication with messages [18,19] and video calls [20]. Another application is supporting rehabilitation through technology that takes place in clinics [21]. Fortunately, patients with MS, especially those younger in age, are willing and able to accept and use technology for self-management and support [18]. In parallel the pharmaceutical industry adopts and utilises digital biomarkers, i.e., metrics provided by technology as opposed to traditional medical assessments and tests, in clinical trials to expedite execution and drug delivery. Specifically, the RADAR-CNS (The RADAR-CNS Project: https://www.radar-cns.org/, accessed on 13 September 2021) utilizes an integrated platform to collect remote monitoring technologies such as sensors and apps with various applications in Central Nervous System (CNS) ailments, including MS. Yet, there is still potential in extracting features and interpreting data for clinician decision making.

Our approach aims for holistic monitoring of multiple aspects of life coupled with event detection through data interpretation to support clinician decision making. The platform integrates comfortable off-the-shelf sensors that provide physical activity, sleep monitoring and heart rate, while analysis extracts health-related problems related to the domain of movement, sleep quality and stress. Through visualizing the sensor data and the detected events, clinicians can take quick decisions pertaining to interventions and follow-up.

### 2.2. Semantic Web Technologies

Over the past few years, considerable attention has been given to the use of Semantic Web technologies in various application domains as they have been proven to be an efficient means of tackling challenges associated with the description, integration and interoperability of information [22]. The rationale is to convert unstructured and semi-structured data and knowledge into interlinked graphs of resources (Knowledge Graphs) with explicit semantics based on W3C standards, like RDF(S) and OWL 2 [11]. The ability to formally capture the intended semantics as symbolic models allows both human experts and machines to interpret unambiguously the information and to further enrich the knowledge graphs using symbolic inference mechanisms, such as native Description Logics inference [23] and rule-based reasoning [24,25,26]. In the rest of this section, we give an overview of key Semantic Web technologies and standards and we describe the related work on the domain of behavioural monitoring using Semantic Web technologies.

#### 2.2.1. Knowledge Representation and Ontologies

An ontology is defined as a set of statements that describe a domain of interest. The main purpose of these descriptions is to prevent misunderstandings, ensuring that the developed solutions and services follow a uniform manner of exchanging information with a certain behaviour (https://www.w3.org/TR/owl2-overview/, accessed on 13 September 2021).

Ontologies have a strong theoretical background, while their expressivity depends on the knowledge representation language that is used to define the models. The Web Ontology language (OWL/OWL 2) [11] has been widely used by the community for defining and sharing ontologies. The theoretical background and semantics of OWL is strongly influenced by Description Logics (DL [23]). Key modelling notions include: (a) axioms, basic facts that can be modelled in OWL, (b) entities, which represent real-world entities, and (c) expressions, which combine entities in order to form complex statements and descriptions. In practice, an OWL model consists of a set of DL axioms (concept inclusions (K⊑T), role inclusions (L⊑M), concept assertions (K(a)) and role assertions (L(a,b)), where *K*, *T* are concepts, *L*, *M* are roles and *a*, *b* are instances.

Reasoning is used in order to make logical derivations, capitalising on the formal semantics of the ontology language. Examples of reasoning services include [23]: class membership, class equivalence, consistency checking, instance classification and realisation. Among other solutions, such as native DL reasoners, symbolic rule-based frameworks [24] have been used in many domains to derive additional relations and enrich the underlying models. Such systems are able to derive relations that are not supported by the standard semantics of the ontology language, like temporal reasoning and structured objects [27,28]. The main concept is to use an existing rule formalism, such as SWRL [29], SPIN [30] and SHACL Rules [15,31], to define custom logic on top of the knowledge graphs describing the conditions that drive the derivation of complex events and situations, expressing richer semantic relations coupling ontological and rule knowledge.

#### 2.2.2. Modelling and Interpreting Activities and Behaviours

Several ontology-based modelling and reasoning frameworks have been developed to cope with challenges relevant to domain modelling, context interpretation and knowledge sharing, with a great number of work focused on home-based activity settings. When it comes to knowledge-driven frameworks, there are mainly two directions: frameworks that make use of native ontology reasoning, and hybrid approaches that extend native ontology reasoning with rules.

Under the first paradigm, knowledge representation formalisms are used for modelling activities explicitly by domain experts. Different profiles of OWL, such as OWL 2 DL, have been widely used within the community for defining domain knowledge, using the underlying semantics (e.g., DL axioms) to capture the modelling requirement of the application domain. Such an approach is presented in [32] where an integrated ontology-based approach is followed for activity modelling (objects and activities), exploiting logical semantic reasoning for activity recognition. The main goal is to support machine understandability through high-level interoperability and intelligent processing. A similar approach is followed in [33,34,35], where complex activities are recognised based on subsumption reasoning. In [35], well-defined symbolic knowledge provides the logic to derive the most probable activity that a user performs among a set of candidates that are identified by statistical methods. In [34], a knowledge-driven approach for real-time, continuous activity recognition is proposed, using ontologies for explicit context and activity modelling, while semantic reasoning performs activity classification. Finally, Riboni, D. et.al [33] presents a system architecture that integrates a novel OWL 2 activity ontology and reasoning modules for sensor data aggregation and activity inferencing. KnowSense [36] has been proposed to support monitoring of activities of people with dementia in controlled environments. Everyday activities are modelled using OWL 2, while a reasoning mechanism identifies activities and problems in different stages of the disease, assisting the clinical evaluation. Okeyo, G. et.al [37] tackles the challenges of real-time continuous activity recognition as sensor data segmentation. The authors present a novel approach to define a dynamic segmentation model, based on the notion of varied time windows, following a knowledge-driven activity recognition algorithm based on ontologies. iKnow [38] tackles the same problem by introducing the notion of telicity in recognising interleaved activities, implementing an ontology-based meta-interpretation layer.

Under the second paradigm, ontologies and rules are combined [24,28] to overcome the expressive limitations of OWL in modelling, aggregating, linking and recognising the monitoring context. These limitations are mainly relevant to features that have been restricted in order to retain the decidability of the language. For example, temporal reasoning and structured objects [27,28] are not supported by the standard ontology semantics. SWRL [29] has been used in a substantial number of relevant solutions, aiming at increasing the interoperability of rule-based systems from the Semantic Web perspective. OWL is combined with RuleML (http://wiki.ruleml.org/index.php/RuleML_Home, accessed on 13 September 2021), providing reasoning capabilities beyond the ones supported by the DLs. For example, Okeyo, G. et al. [39] uses SWRL to model activities and their temporal relations, based on [40]. A framework for behavioural analysis with SWRL is presented in [41,42], generating reminders and applying strategies to promote a healthier lifestyle. HABITAT [43] aims to improve the quality of life, supporting elderly through their main daily life activities. It uses the SPARQL Event Processing Architecture (SEPA) to perform SPARQL updates and queries on the underlying RDF graph, enabling to monitor the daily behaviour of people that face problems in home settings due to ageing or illnesses. HEARTDROID [44] uses a rule inference engine for Android mobile devices. It allows the definition of semantic annotations of its components in order to increase intelligence and transparency of the model and the reasoning services. In [45], multimodal historical and real-time sensor data are acquired, feeding a rule-based activity recognition system. The system corrects erroneous sensor data through simple heuristics and cross-validation against other modalities, achieving indoor localisation and activity recognition through the SPHERE ontology.

As described in [46], the modelling approaches fall into two categories: knowledge-driven approaches, as the ones presented above, leveraging logical and knowledge representation formalisms and reasoning, and data-driven approaches that exploit machine learning. Both strands have their strengths and weaknesses. In the knowledge-based approaches, domain and common sense knowledge is directly incorporated into activity models. This works particularly well in cases where there are no data for training. In addition, the activity models can be reused across domains and users or even to be manually refined, according to specific behavioural aspects. However, the logic-based approaches are not robust against noise and uncertainty and require carefully crafted rules. On the other hand, data-driven approaches [47,48,49,50] work better when large collections of training sets are available, being generally robust to noisy, uncertain and incomplete data. However, the models are not reusable, compared to knowledge-driven solutions, having limitations on the amount of common sense knowledge that can be incorporated. Finally, one more category of behavioural modelling could be considered the hybrid approaches that combine the knowledge- and data-driven approaches, such as [51,52,53,54], focusing on increasing the performance of ontology-based activity recognition, and vice versa, through data-driven pre-processing (e.g., the learning of activity models).

Our framework follows the knowledge-driven paradigm where activity and behavioural models are predefined. Our solution has been primarily used in the domain of Ambient Assertive Living, providing integrated information to clinicians about patient behaviour and health status, while supporting individuals to improve their lifestyle and adopt healthier living routines. In this context, the logic that underpins the predefined behavioural patterns and the detection of abnormal situations is given by the clinical experts, allowing them to configure it on demand, guiding and controlling monitoring to take into account certain behavioural aspects. As such, clinical experts can easily and directly adjust the logic that underpins the detection of certain situations, according to monitoring goals and user background knowledge. In addition, the patterns can be reused among individuals or even further refined, without needing to rebuild models or collect additional data.

This work capitalises on and considerably extends previous work of ours in the domain [55]. More specifically, we present here in detail the specifics and logic of the underlying conceptual model that is used to represent observations, introducing the notion of situations and enriching the interoperability of our KGs using existing modelling standards. A new rule-based extension is presented, using SHACL as the underlying rule language for detecting problematic situations. Finally, we present user- and system-centred evaluation results.

## 3. Overview of the Framework

The aim of the framework is to support the monitoring of problematic areas of daily living, utilising wearable sensors and mobile devices for feedback and intelligent analysis in an Ambient Assisted Living context. To this end, it integrates state-of-the-art technologies on knowledge representation, data integration, access and visualisation of information, providing to clinicians, patients and caregivers with the information and feedback necessary for the detection and management of lifestyle and health-related problems. Through the extensive use of Semantic Web technologies and frameworks, we provide an intelligent, easy-to-adapt framework for personal and clinical use, using structured knowledge graphs to represent both behavioural (observations) and personalised health knowledge in the form of aggregation rules. Coupled with adaptive user interfaces, we support, on one hand, individuals to improve their lifestyle and adopt healthier living routines, and, on the other hand, health experts to monitor specific behavioural aspects and design personalised interventions and care plans.

**Figure 1:**
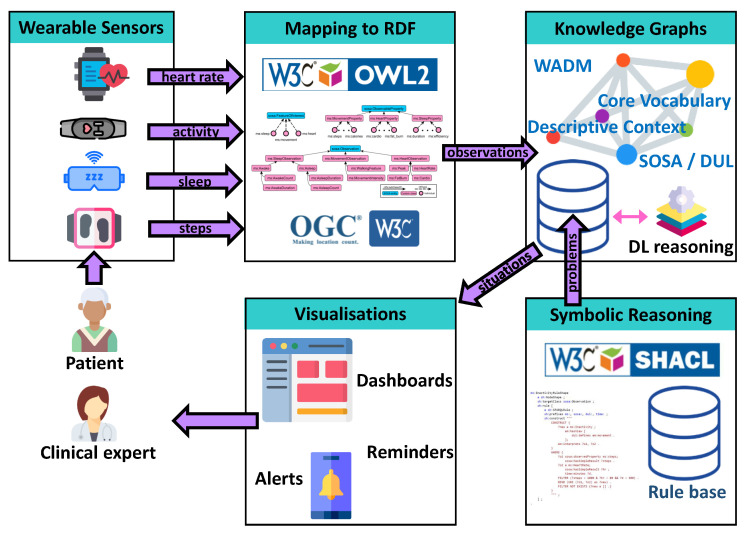
The conceptual architecture of the proposed framework.

The implementation of the framework is based on well-established Semantic Web technologies, standards and ontologies. To this end, it can be easily reused and adapted to different application domains and monitoring requirements. As we explain in Section 4.2, the framework promotes portability and it can be easily adapted to different monitoring traits, according to clinical goals and user preferences. The main building blocks of our approach are depicted in Figure 1. More specifically:

- Wearable Sensors: The input to our framework is data collected by users using various wearable sensors. The framework does not impose any restriction on the modalities that can be integrated, provided that the underlying ontologies contain the necessary constructs to support their representation. The framework currently supports the representation of information about sleep attributes, steps, heart rate and other activity-related measurements. The sensor used currently is Fitbit Charge 3 (https://fitbit.com. accessed on 13 September 2021), which provides steps as an indication of physical activity level, sleep staging and heart rate, collected through the Fitbit smartphone app and extracted from Fitbit’s cloud API (after authorised by the user using OAuth).
- RDF Mapping and Knowledge Graphs: The incoming data are then transformed into RDF observations, generating the structured RDF Knowledge Graphs. The conceptual model follows the SOSA ontology, which has been extended to meet the observation types supported by the implementation. A general-purpose semantic graph database is used (https://graphdb.ontotext.com/, accessed on 13 September 2021) to persist the Knowledge Graphs, interfaced with a DL reasoner [56] to handle the semantics of the schema.
- Symbolic Reasoning: We follow a knowledge-driven approach, using a set of preconfigured rules to detect problematic situations and activities of interest. The detection logic, which follows clinical guidelines and user preferences, is encoded in a set of SHACL SPARQL Rules (see Section 4.2) that run on top of the Knowledge Graph and generate problems.
- Visualisations: A web application has been designed and developed to serve the needs of clinical experts, based on their requirements. The dashboard visualisations design takes into consideration performance, acceptance, clinical and therapeutic value characteristics, based on design choices of previous works in other eHealth fields [57].

A modular, Service-Oriented Architecture was used to integrate multiple kinds of wearable smartwatches, wristbands and any type of smart home sensor in an extensible manner. Most devices available in the market provide their data through their own well-defined Cloud APIs or Software Development Kits (SDKs). For each device integrated, a “connector” module is developed that leverages according APIs or SDKs to retrieve device data and in turn expose it through its own secure API accessible to the platform, which stores it in a uniform, semantic format. Platform data become available for further analysis and interpretation, as well as for display in user applications through semantic search and consumption protocols (SPARQL endpoints).

For the purposes of MS monitoring, we chose to deploy the FitBit Charge 3 smartwatch device due its long battery life and ability to extract sufficient data for the study. The discrimination of awake and sleep stages in this study is entirely based on the Fitbit device and algorithms, which in a recent systematic review are deemed highly accurate, especially in differentiating wake from sleep [58]. The goal of the framework is to extract further health-related problems from the sensor’s data, that are clinically relevant to MS. The connector module developed retrieves intraday data, up to a per minute sampling rate, from FitBit Cloud through secure user authorisation (OAuth protocol) after the end-user (patient) authorises it. Namely, the available data are listed on Table 1. Notably, the wearable does not provide raw accelerometer data but rather pre-processed/aggregated data that can later be used to extract events and patterns. In the following section, we elaborate on the specifics of the framework.

## 4. Semantic Web Approach to Detection

Our solution is based on the use of Semantic Web technologies to support the interoperable representation of the different modalities integrated in our framework and to facilitate their intelligent aggregation and interpretation for activity and behavioural monitoring. More specifically, we have developed a number of OWL 2 ontologies and modelling patterns to capture information at different levels of abstractions, aiming to capture information and background knowledge as interlinked RDF Knowledge Graphs (RKGs). Apart from the advanced connectivity and interoperability that is offered through the use of formal semantics, the RKGs provide also a framework for intelligent data integration, unification, analytics and sharing. This is achieved by implementing a symbolic reasoning layer on top of the RKGs consisting of a set of inference rules that aggregate and combine the underlying descriptions and elicit an understanding of situations.

In the following sections, we describe the schema and the data semantics of the RKGs, and we elaborate on the specifics and capabilities of the symbolic reasoning layer.

### 4.1. Semantic Representation and Knowledge Graphs

Conceptually, our framework supports two levels of abstraction through which domain information can be modelled: *observations* and *situations*.

- Observations: The semantics and structure of the various modalities are captured as observations. This involves information about the types of the sensors and the devices used for monitoring, and the attributes of the measurements they produce. SOSA is used as the main building block to describe the measurements.
- Situations: It corresponds to higher level knowledge, providing the constructs to capture the logic for the detection of problematic situations through aggregation rules that combine the available input and generate additional inferences. It also serves as the root for modelling problems and behavioural aspects. DUL [59] is used as the underlying conceptual model, exploiting the alignment of SOSA to the DOLCE UltraLite upper ontology (https://www.w3.org/ns/ssn/dul, accessed on 13 September 2021), promoting interoperability with other DUL-aligned ontologies [60,61,62].

In addition to the inherent association of observations with situations that is part of DUL, we implement an additional alignment of our conceptual model with the Web Annotation Data Model (WADM [13]) that describes a structured model and format to enable annotations to be shared and reused across different hardware and software platforms, following a standard and lightweight description model. This additional level of annotation serves as a valuable tool for querying, sharing and reusing the knowledge in domains that follow a simple conceptual model. In the following sections we present the specifics of each abstraction layer, together with examples.

#### 4.1.1. Observations

The measurements that are generated by the various sensors and devices are captured as observations. This involves measurements about sleep (e.g., duration, efficiency), heart-related measurements (e.g., average fat burn rate), activity-related characteristics (e.g., step count, calories burnt) and other modalities, as these are described in Table 1.

**Figure 2:**
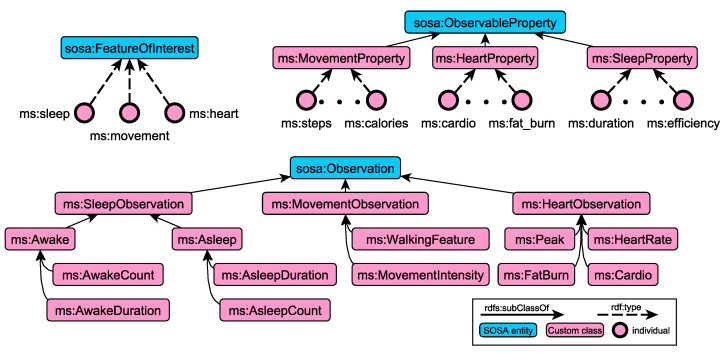
Examples of concepts and instances that have been defined on top of the SOSA conceptual model for capturing domain-specific observation types.

The SOSA ontology is used as the core data model, providing the necessary vocabulary to capture measurements, sensors and observable features. A conceptual model of observation types has been defined to organise observations into hierarchies, capitalising on OWL 2 class subsumption and class equivalence semantics. Figure 2 depicts a subset of the ontological schema we are using to capture information about observations, following the conceptual model of SOSA. The core pattern of SOSA revolves around feature of interests, observable properties and actual observations. A feature of interest captures the notion whose (observable) property is being estimated or calculated in the course of an observation. In our example (Figure 2), this corresponds to sleep, movement and heart features (upper left model), whose properties (upper right model, e.g., steps, fat_burn and duration) are estimated in terms of observations (model at the bottom). Furthermore, in order to assist querying and reasoning processes, observations are organised into a hierarchy of types. It is worth noting that the thresholds (or logic) to generate these observations from the sensor data depend on DL reasoning [63]. Examples of such axioms are given in Table 2. For example, a generic observation instance whose observedProperty is associated with an instance of the HeartProperty and has the feature heart as a feature of interest, is automatically classified as a HeartObservation. In that way, our framework separates the modelling logic from the technical specifications of the incoming observations, enabling the further adaptation and extension of the conceptual schema without requiring any change on the data collection mechanism.

Rich semantic relationships for instance class membership have been defined to support the automated classification of the incoming observations in the hierarchy of observation types. To this end, the definition of the observation classes have been enriched with OWL complex class descriptions (OWL is used in this paper to refer to both OWL and OWL 2 ontologies interpreted under Direct Semantics) using DL axioms. For example, the MovementObservation class is defined as:

(1) $$\begin{array}{c} \mathrm{MovementObservation}\equiv \mathrm{Observation}⊓\exists \mathrm{observedProperty}.\mathrm{MovementProperty}\\ \;⊓\exists \mathrm{hasFeatureOfInterest}.\{\mathrm{movement}\} \end{array}$$

Equation (1) is an example of the complex class expression capabilities of OWL 2 ontologies. More specifically, it uses a class equivalence axiom (≡) that defines necessary and sufficient conditions for instance class membership, i.e., restrictions that should be satisfied in order for an instance to belong to the class MovementObservation. The restrictions are defined as an intersection (⊓) of multiple existential restrictions (∃). More specifically, the first existential restriction defines that the instance should define at least one property assertion of type observedProperty, whose value should belong in the class MovementProperty. Similarly, the second existential restriction defines that there should be at least one property assertion of type hasFeatureOfInterest, whose value should be the instance movevent. These complex class expressions are used throughout the paper and in Table 2. All in all, the class equivalent concept in (1) defines the MovementObservation concept as the set of all observation instances that have at least one observedProperty property assertion that associates the observation with an instance of the MovementProperty concept. At the same time, all the relevant observations should have hasFeatureOfInterest property assertions with the instance value movement. Intuitively, each observation that measures the quality of movement through a movement property, such as steps, is classified in the MovementObservation class.

The complex class descriptions are inherited to the subclasses, following the class subsumption hierarchy, where they can be further extended with local definitions. For example, the WalkingFeature observation class inherits the DL axioms defined in (1) and further enriches its definition with the following axioms:

(2) $$\begin{array}{cc} \mathrm{WalkingFeature} & \equiv \mathrm{MovementObservation}\\ \; & ⊓\exists \mathrm{observedProperty}.\{\mathrm{distance},\mathrm{elevation},\mathrm{floors},\mathrm{steps}\} \end{array}$$

The description in Equation (2) further restricts the observedProperty assertions to contain one of the four observed properties that are associated with walking-related attributes.

**Figure 3:**
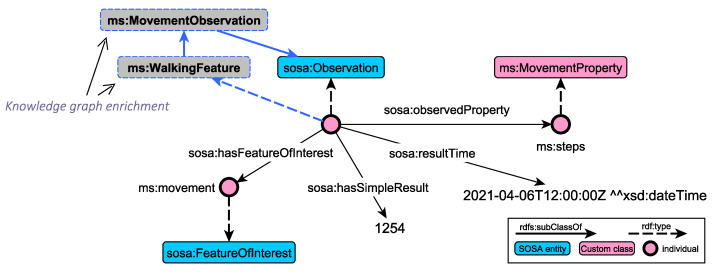
Example classification of an observation instance. Based on complex class description axioms in the observation hierarchy, the instance is automatically classified in the MovementObservation and WalkingFeature classes through ontology reasoning (highlighted in blue).

As an example, consider the observation instance in Figure 3 that measures the quality of movement through the number of steps. More specifically, the figure illustrates the excerpt of the RDF knowledge graph that captures the basic concepts of the SOSA conceptual model. We can observe that the observation instance (type of sosa:Observation) acts as a container that links together other entities of the conceptual model, like the observed property (steps), feature of interest (movement), as well as additional descriptive information relevant to the temporal extension and actual result. One unique feature of the RDF model, and thus of the RDF knowledge graphs, is the possibility to dynamically change the underlying schema, based on domain knowledge and inference rules. As such, the knowledge graphs can be further enriched with additional relations, enabling the capturing of additional semantics. In our domain, using OWL 2 reasoning, the observation is classified as an instance of the MovementObservation class (due to Equation (1)). Moreover, due to Equation (2), the observation finally belongs to the class extension of WalkingFeature. Similar logical axioms have been defined for the other observation types, fostering the automated classification of incoming observations using ontology reasoning. Table 2 presents the set of axioms that is currently supported for basic observation types.

#### 4.1.2. Situations

At a higher level of abstraction, our conceptual model supports the definition of situations. Compared to observations that capture asserted information (e.g., sensor measurements), situations encapsulate aggregated views of state of affairs, such as behaviours and problems, that are derived by intelligently analysing and aggregating contextual information. As we describe in Section 4.2, a symbolic reasoning layer is responsible for aggregating and interpreting the asserted knowledge, deriving situations of interests based on user preferences and clinical guidelines.

**Figure 4:**
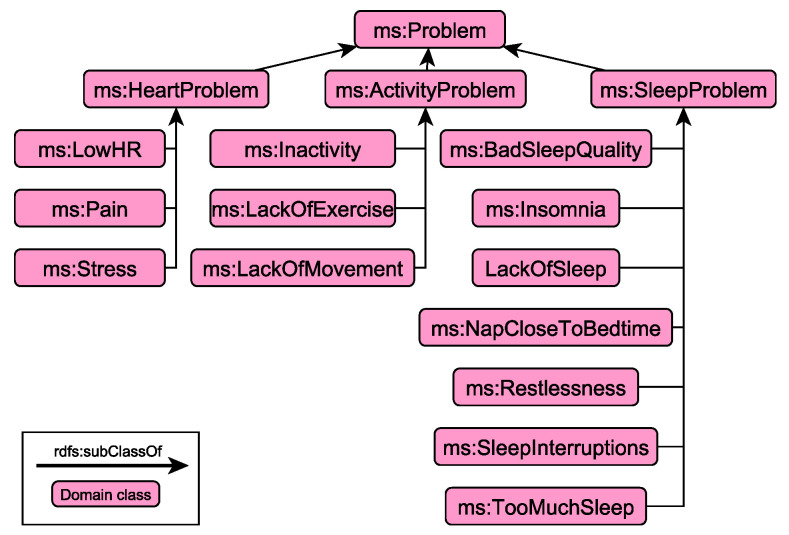
Example hierarchy of the Core Vocabulary relevant to problematic situations whose monitoring and detection is currently supported by the framework.

The conceptual model of situations consists of two parts: the *Core Vocabulary* and the *Descriptive Context*. The Core Vocabulary models the specifics of the domain in terms of behavioural aspects and problems. Figure 4 presents an example of the hierarchy that supports the modelling of problematic traits relevant to sleep, activity and heart-related characteristics.

More specifically, following the same conceptual modelling principles described in the previous section regarding observations, problems are further organised into specific categories, allowing user to browse more efficiently the underlying knowledge graphs, while the dashboards presented in Section 5 have direct access to specific type of information, easing the development of the necessary interface to get access to specific parts of the underlying knowledge graphs. As illustrated in Figure 4, problems are categorised into heart-, activity- and sleep-related ones, according to the type of observations used to derive these problematic situations. In contrast to the observation hierarchy that the classification of observation instances is based on DL reasoning, the different types of problems are derived through rule-based symbolic reasoning (see Section 4.2).

On the other hand, the Descriptive Context acts as the “semantic glue” between observations and situations, providing the definitions and a formal structure for describing implicit and explicit concepts and relationships between the asserted and inferred context. To this end, the implementation of the Descriptions and Situations (DnS [14]) pattern in DUL is used, exploiting the already defined DUL-SSN alignment (https://www.w3.org/ns/ssn/dul, accessed on 13 September 2021).

The OWL encoding of DnS assumes DOLCE as a ground top-level vocabulary. The DOLCE+DnS Ultralite (DUL) is a light version, which provides simplifications and improvements of some parts of DOLCE and DnS. Its purpose is to provide a set of upper level concepts that can be the basis for easier interoperability among many middle and lower level ontologies. More specifically, DnS tries to capture the notion of “Situation” out of a state of affairs, with their interpretation being provided by a “Description”:

- Situation: A set of domain entities that are involved in a specific pattern instantiation.
- Description: Serves as the descriptive context of a situation, defining the concepts that classify the domain entities of a specific pattern instantiation, creating views on situations.
- Concepts: Classify domain entities describing the way they should be interpreted in a particular situation.

The basic implementation of the DnS pattern in DUL allows the relation of situations (dul:Situation) and descriptions (dul:Description) with domain concepts (dul:Concept). More specifically, a situation describes the entities of a context and satisfies (dul:satisfies) a description. The description in turn defines (dul:defines) concepts that classify (dul:classifies) the entities of the situation, describing the way they should be interpreted.

**Figure 5:**
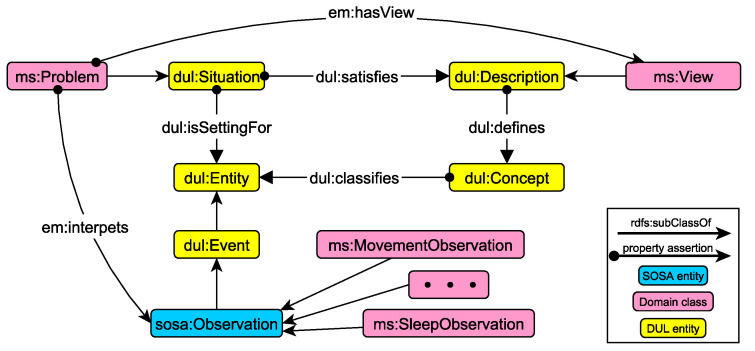
The core DnS pattern in DUL with the domain extensions and adaptations that formulate the Descriptive Context.

The Descriptive Context in our framework is defined as an adaptation of the DnS pattern, reusing the DUL-SSN alignment and defining the necessary domain concepts to facilitate the interlinking of observations with problematic situations. Figure 5 presents the domain extensions of the pattern. More specifically, the classes in yellow represent core classes of the DnS pattern, while our class extensions are represented in red. Intuitively, problems are situations that can be interpreted in terms of view on specific observations. This conceptual model is in line with the DnS model, where situations are linked to description that point to concepts and entities. In that way, by following a foundational upper level ontology and general-purpose pattern (DUL and DnS), we promote interoperability of the underlying knowledge graphs, as they can be easily aligned to existing standards, such as SOSA.

More formally, the alignment and adaptation to the DnS pattern is given by the following DL axioms:

(3) $$\begin{array}{cc} \mathrm{ms}:\mathrm{Problem}⊑ & \mathrm{dul}:\mathrm{Situation}⊓\;\exists \mathrm{em}:\mathrm{hasView}.\mathrm{ms}:\mathrm{View}\\ & ⊓\;\exists \mathrm{em}:\mathrm{interprets}.\mathrm{sosa}:\mathrm{Observation} \end{array}$$

(4) $$\begin{array}{cc} \mathrm{ms}:\mathrm{View}⊑ & \mathrm{dul}:\mathrm{Description}⊓\;\exists \mathrm{dul}.\mathrm{defines}.(\mathrm{dul}:\mathrm{Concept}\\ & ⊓\;\exists \mathrm{dul}:\mathrm{classifies}.\mathrm{sosa}:\mathrm{Observation}) \end{array}$$

(5) $$\begin{array}{c} \mathrm{em}:\mathrm{hasView}⊑\mathrm{dul}:\mathrm{satisfies} \end{array}$$

(6) $$\begin{array}{c} \mathrm{em}:\mathrm{interpets}⊑\mathrm{dul}:\mathrm{isSettingFor} \end{array}$$

(7) $$\begin{array}{c} \mathrm{sosa}:\mathrm{Observation}⊑\mathrm{dul}:\mathrm{Event} \end{array}$$

Problems are captured as situations that are associated with views and interpret a set of observations (axiom Equation (3)). Each view defines one or more domain concepts that classify the observations relevant to the detected problem (axiom Equation (4)). Certain properties (subproperties of DUL relations) are used to associate problems with views (axiom Equation (5)) and observations (axiom Equation (6)). Finally, observations are defined as subclasses of DUL events (axiom Equation (7) is part of the DUL-SSN alignment).

**Figure 6:**
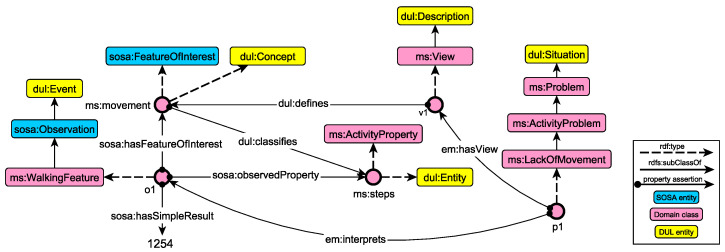
Example instantiation of the Descriptive Context, semantically describing a problematic situation about lack of movement together with the associated observation.

Figure 6 presents an example instantiation of the pattern. The knowledge graph captures information about the detection of an activity problem p1 about the lack of movement. Following the pattern of the Descriptive Context, p1 represents a problematic situation that has a view and interprets one or more observations. The view v1 (em:hasView property assertion) defines movement as a concept (through the dul:defines property assertion), which in turn classifies steps. At the same time, p1 interprets the o1 observation, which is associated with the movement and steps instances through SOSA property assertions (sosa:hasFeatureOfInterest and sosa:observedProperty, respectively). As such, o1 is conceptually annotated with DUL- and SOSA-related concepts, enabling its semantic interpretation as a problem, attaching rich information about the concepts, entities and relationships that underpin the observed property and feature of interest. It is worth noting that such RDF graphs are generated through symbolic reasoning, as we describe in the next section.

The alignment and mapping to other conceptual models and datasets is feasible, promoting data exchange, knowledge sharing and reuse. Table 3 presents the conceptual mapping of the Descriptive Context (Figure 5) to the Web Annotation Vocabulary [64] that defines the RDF classes, predicates and named entities used in WADM. In WADM an annotation has 0 or more bodies (oa:hasBody), which encapsulate descriptive information, and 1 or more targets (oa:hasTarget) that the bodies describe. The mappings define problematic situations as annotations, with views representing the annotation bodies and the interpreted concepts providing the targets of the annotations, conceptually aligning the two models.

### 4.2. Symbolic Reasoning

Given an RDF graph of observations, as these are defined in Section 4.1.1, the goal of symbolic reasoning is to meaningfully aggregate, correlate and interpret the collected information to elicit an understanding of the situation and detect health-related problems, according to experts domain knowledge.

The symbolic reasoning layer in our solution is defined with SPARQL, executing iteratively a set of SPARQL CONSTRUCT graph patterns to derive inferred RDF triples and enrich the Knowledge Graphs with problematic situations, as these have been described in Section 4.1.2. In order to promote interoperability and further foster the interaction of OWL Knowledge Graphs with other emerging Semantic Web standards, the SPARQL graph patterns are wrapped as SHACL Rules [15]. SHACL [31] is a W3C recommendation recently introduced to define properties of RDF datasets. SHACL allows the definition of constraints that can be validated against RDF graphs. Inference rules are also supported (SHACL Rules) to form a light-weight RDF vocabulary for the exchange of rules that can be used to derive inferred RDF triples from existing asserted triples.

Table 4 presents a subset of the rules that have been encoded as SHACL rules. These rules were derived after several iterations with the doctors, psychologists and patients in order to clearly define the monitoring context and the problematic situations, according to profile information. The rules define the upper and lower limits to filter out observations that do not match the defined logic. The numerical values of the limits were decided after consultation of the clinicians and the patients. It should be noted that different rules and thresholds are used for each monitored individual, according to preferences and clinical goals. For example, in the first rule, the threshold for detecting insomnia was set at 1800 s, taking into account the sleep habits of that user. In addition, to conclude that the patient needs to exercise more, their steps are limited to less than 8000 in one day.

**Figure 7:**
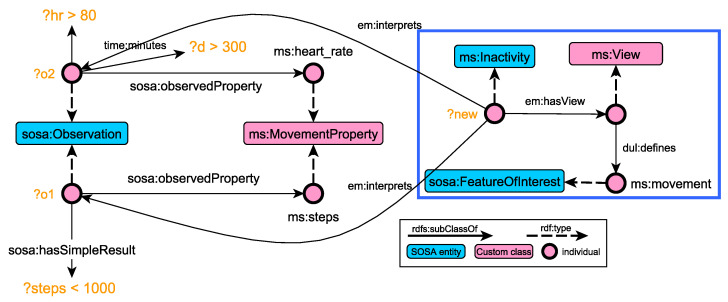
Graphical representation of the SHACL SPARQL rule for inactivity problems. The RDF graph inside the blue box is generated after the successful checking of the conditions highlighted in orange.

Figure 7 presents an example of a SHACL SPARQL rule for detective inactivity problems (Table 4). The rule also illustrates the capabilities of the modelling layer, as far as interoperability is concerned. The rule actually integrates three conceptual models: SOSA, for matching observations, DUL, for implementing the DnS pattern, and the custom extensions described in this paper (e.g., em:Inactivity). As such, the generated knowledge graphs can be queried using well-established vocabularies, while it can be easily linked with monitoring frameworks that follow similar conceptual models [60,61].

## 5. Clinician Dashboard and Use Case Applications

This section presents the clinician dashboard, the web app that clinicians use to observe progress and care and decide on interventions, as well as a use case application on actual patients.

### 5.1. The Clinician Dashboard Web Application

The clinician dashboard is an end-user application with Graphical User Interfaces (GUIs) that aims to provide clinicians with visualisations of both raw data and detected events to enable informed decisions. Clinicians can select periods of time and view graphs and events. Thus, they can detect lifestyle trends and patterns in time and suggest tailored interventions. In turn, they can monitor adherence, behavioural change and positive impact in various areas. Particularly for MS, the dashboards aim to show a holistic view of both physical and mental health aspects of wearable data and tailored event detection.

The clinician dashboard is implemented as a web application using open source frameworks and libraries, mainly the well-established and modern Python Django web framework. Responsive design ensures adaptation to both mobile phones and tablets and large computer screens. Communication with analysis subsystems and the knowledge base is performed via secure APIs.

**Figure 8:**
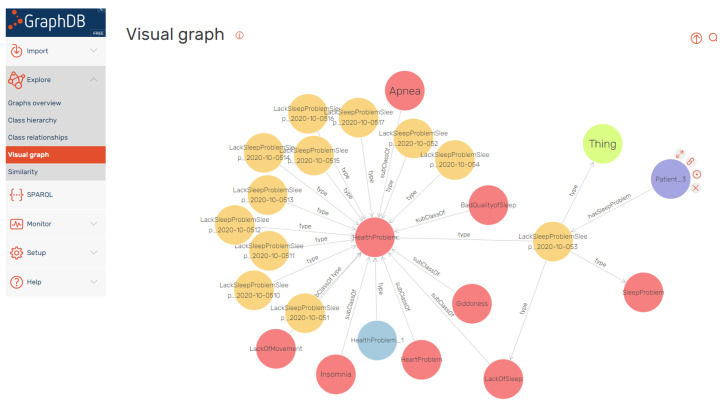
Direct browsing of the Knowledge graph of observations and problems. The visualisation has been generated using the visual graph capabilities of the GraphDB repository. The nodes represent RDF resources (both classes and instances). The color and size of the nodes depend on internal statistics of the repository. For example, the size of the nodes reflects the importance of the node by RDF rank. Intuitively, nodes linked with a subclass relation represent classes, and the end of type arrows points also to classes. In all the other cases, the nodes refer to instances in the KB.

Clinical experts can browse directly the knowledge graphs, through a user-friendly rendering service provided by the underlying triple store. Figure 8 depicts the dashboard that allows user to expand specific nodes of the graph, acquiring associating context. The figure depicts the graph associated with lack of sleep incidents for a certain user.

**Figure 9:**
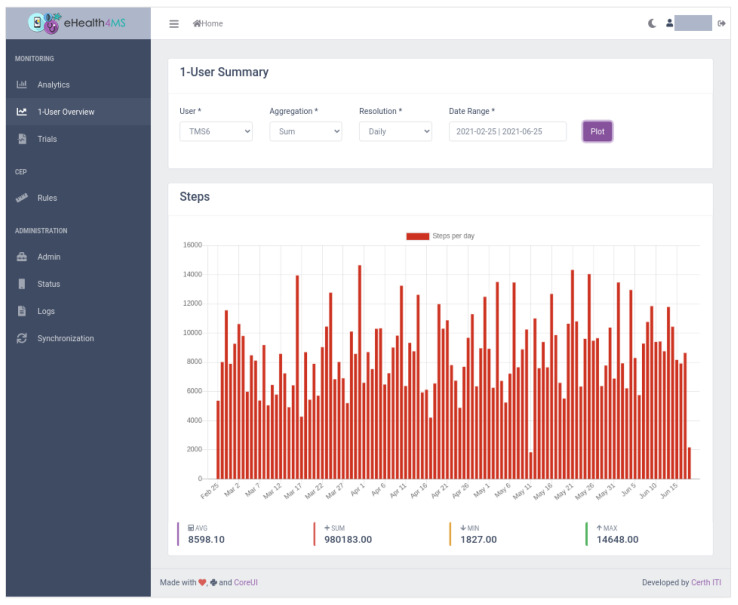
Clinician Dashboard showing visualizations for clinicians: Number of daily steps for user TMS6.

**Figure 10:**
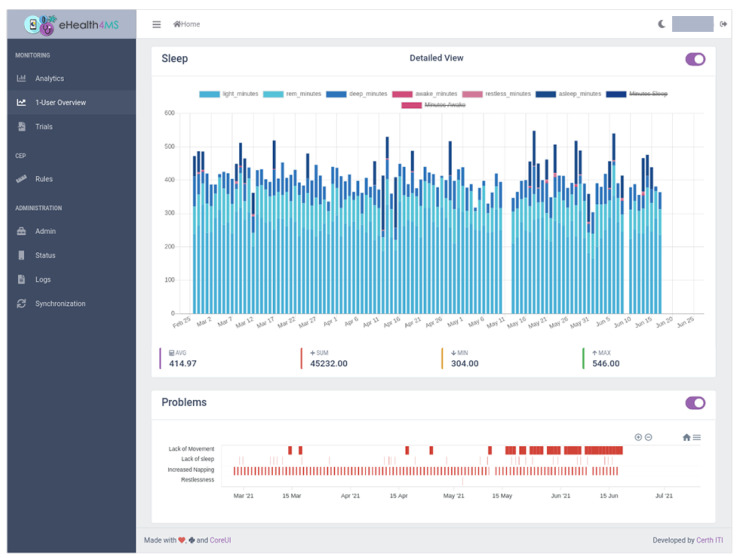
Clinician Dashboard showing visualizations for clinicians: Daily sleep metrics and detected events for user TMS6.

**Figure 11:**
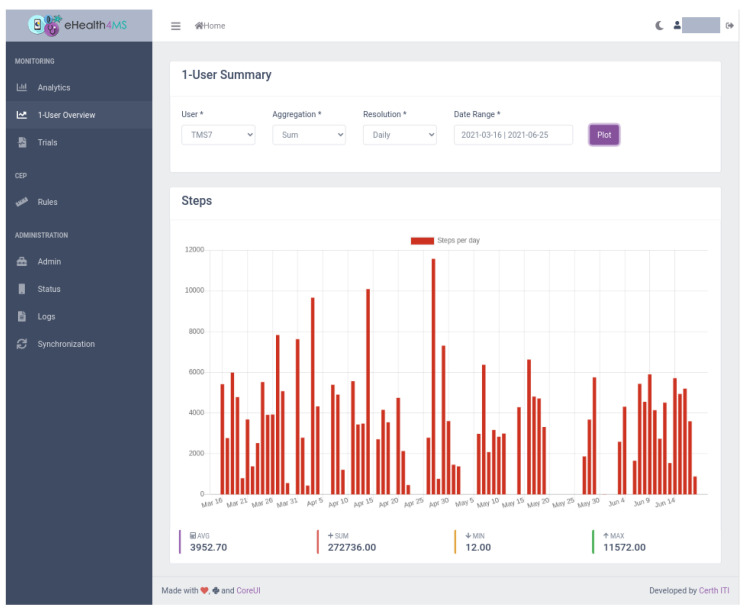
Clinician Dashboard: Number of daily steps for user TMS7.

**Figure 12:**
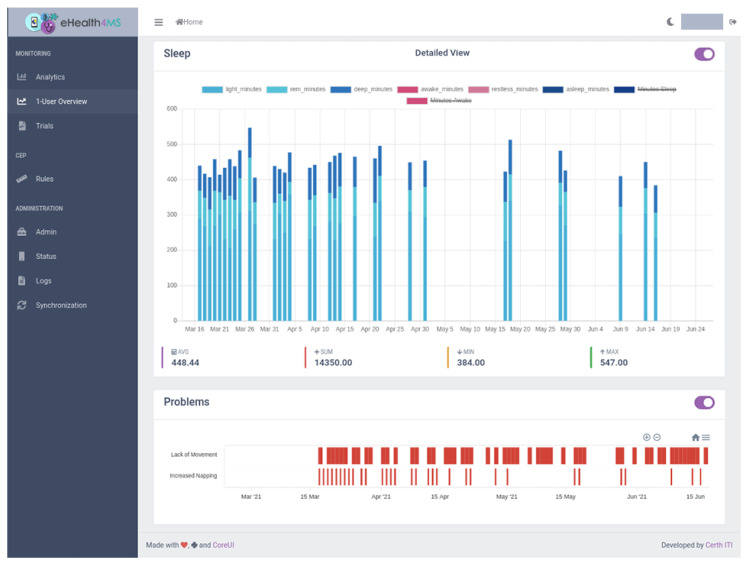
Clinician Dashboard: Daily sleep metrics and detected events for user TMS7.

The dashboard’s main view for clinicians is “1-User Summary” (Figure 9, Figure 10, Figure 11 and Figure 12), which shows steps, sleep, heart rate and problems (detected events) for the selected user, aggregation method (sum or average), resolution (per minute, hour, day, week, month, year) and date range. Steps and heart rate are shown in bar charts, while sleep charts also contain segments according to sleep stage for each interval. Detected events are shown on a timeline. Thus, clinicians can observe trends in time and further zoom in or out by changing parameters and explore overall patterns and investigate occurrences and context to suggest interventions.

### 5.2. Use Case Application

In order to demonstrate the usefulness of the Clinician Dashboard and the underlying system, we recruited two patients with MS in Thessaloniki, Greece. The bioethics committee of the Greek Association of Alzheimer’s Disease and Other Dementias has approved the study with regards to data collection and written informed consent was provided by all participants. After several months of data collection, the raw sensor data and events automatically detected by the system are visible on the Clinician Dashboard application. As their demographic and medical information are not relevant to the use case scenarios demonstrated here, they are not disclosed. For privacy reasons, the users are pseudonymized as “TMS6” and “TMS7”.

After applying the rules of Table 4 for all users, a use case is defined as follows: a clinician wishes to review data for a patient to discover patterns and problems and to decide on which area to focus for follow-up and intervention. Two scenarios are presented, one for each patient. Observations based on the sensor data are reviewed at first and then the additional events are detected by the system. For both scenarios, the parameters chosen were aggregation by sum and daily resolution.

#### 5.2.1. Scenario 1: User TMS6

- The clinician selects the user “TMS6” from the drop-down box, the sum aggregation method, resolution per day and a date range from end of February, when the patient was recruited, to today, end of June. In a few seconds, the dashboard loads the data in the Knowledge Base (KB).
- First, it shows the total sum of steps per day (Figure 9). From the raw sensor data the overall behaviour is inconclusive: there seems to be a lot of variance, a high number of steps on some days and a low on others. The dashboard already adds some value by showing the average, minimum and maximum values below the chart. The average for this user is above 8000 steps which means that he is moving adequately (more than 5000 steps). Still, we do not know how often they hit the threshold and whether there is a pattern forming here.
- Moving further down the page, we view the sleep data as detected by the sensor (Figure 10). The user seems to be getting enough sleep most days minus a few outliers. Furthermore, the dashboard analytics help to ensure that the total duration of awakenings is low (pink areas in the chart) and that the averages seem adequate. However, again, the human eye alone can not distinguish a pattern or a problem emerging. Furthermore, despite the duration of sleep and awakenings appear sufficient, it is hard to tell whether the number of awakenings and naps is problematic.
- Finally, the clinician views the Problems detected by the system. A “Lack of Movement” instances show up sporadically in the first couple of months but intensify in the last month to almost every day. That flags a potential relapse that can link to physical or mental hindrances to the patient, so the clinician will need to follow up with the patient on their status and a potential intervention.
- Regarding the other problems detected by the system, “Lack of Sleep” is only sporadic and “Restlessness” is very rare, which confirms the adequate sleep duration and the low duration of awakenings in the raw sensor data observed before.
- The most common problem with the patient seems to be “Increased Napping”, which means that although the total sleep duration for a day is adequate, they accumulate over 3 naps per day, which indicates a tendency for lethargy that needs to be addressed by finding the underlying causes and suggest an intervention. This observation could not be easily accessible through raw sensor data but the system immediately flags this as a problem.

#### 5.2.2. Scenario 1: User TMS7

- The clinician selects the user “TMS7” from the drop-down box, the sum aggregation method, resolution per day and a date range from the end of February, when the patient was recruited, to today, the end of June. In a few seconds, the dashboard loads the data in the KB.
- Looking at the visualised raw sensor data for steps per day (Figure 11), the overall behaviour is inconclusive: adequate movement seems to be achieved in very few days, while for most there is little to no movement. The dashboard analytics help clear the picture by showing the average number of steps being indeed low (around 4000).
- The lack of sleep data (Figure 12) indicates a lack of adherence in the sense that the user is probably not wearing the sensor during night sleep in days where there are recordings for steps. The clinician will follow up on this issue and investigate the reasons. Other than that, the sleep data quality shows enough sleep in most days and close to no awakenings.
- Finally, the clinician views the problems detected by the system. Interestingly enough the “Lack of Movement” problem shows up after an initial period, something that was barely visible to the bare human eye examining the steps chart previously. The same applies to “Increased Napping” which seems to appear on the same period and may be linked to a behaviour of not moving and napping at home. The clinician will follow up on lack of movement causes and interventions.
- No other problems are detected by the system, which confirms that awakenings, restlessness and sleep quality is high with the exception of napping.

In both instances, the scenarios highlight the added value that the visual dashboard adds to the raw sensor data view, and most importantly, the value of the problems detected by the system in terms of efficiently pinpointing patterns, behaviours and potential relapse.

## 6. Performance and Clinician Perspectives

Given the lack of a golden standard ground truth, to the best of our knowledge, regarding problem and health-related event detection of such symptoms from wearable sensor data in MS, we carried out a performance evaluation, to attest the scalability and applicability of the system, as well as an exploratory research study with clinical experts to investigate their perspectives on the usefulness of the framework.

### 6.1. Performance Evaluation

In order to apply the system in actual use, the analysis tasks are assigned to routines on a server to be triggered at periodic intervals (chronjobs) or at the presence of new data (e.g., upon a new sleep session entry). Therefore, the clinician dashboard takes only a few seconds to load several months of worth of data and analysis outcomes (events) for a given user. Although this processing load is postponed to certain intervals, it still may scale as the users and the number of objects, i.e., sensor readings to ingest in the KB, increase. It may also scale according to the number of new knowledge to ingest in the KB according to how many rules are fired per a given instance of existing objects.

To evaluate scalability performance, we carried out two tasks using a synthetic dataset generated out of real data, extended for quantity, when needed:

1. Task 1: Measure the time for ingestion for an increasing number of objects in the KB.
2. Task 2: Measure the time and the number of objects generated for an increasing number of existing objects in the KB and for three different rules.

**Figure 13:**
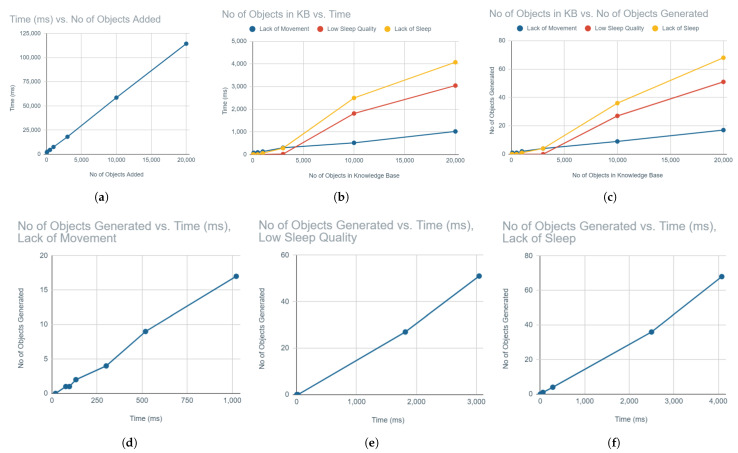
Scalability of the system in different context. (**a**) Time for an increasing number of objects to ingest in the KB. (**b**) Time for an increasing number of objects in KB for each rule. (**c**) Objects generated for an increasing number of objects in KB for each rule. (**d**) Time for an increasing number of objects generated for “Lack of Movement”. (**e**) Time for an increasing number of objects generated for the “Low Sleep Quality” rule. (**f**) Time for an increasing number of objects generated for the “Lack of Sleep” rule.

In Task 1, the goal was to observe the scalability of the KB itself as the load of objects to insert increases. Table 5 shows the measured times for the increasing load. As seen on Figure 13a, the time to add objects grows in a linear relation to the number of objects to be added, which is optimistic for performance.

In Task 2, the goal was to measure how much this load is actually expected to grow under the current expectations, i.e., the growing objects to check and new knowledge to ingest. Given that all rules are similar in terms of adding one new object (problem event) when their conditions are activated, the varying factor are the different conditions. Therefore, we chose three representative rules to detect “Lack of Movement”, “Low Sleep Quality” and “Lack of Sleep” and performed the task for each of them for an increasing number of objects in the KB to check. Table 6 shows the measurements.

To visualise the outcomes, first we consider execution time in relation to the number of objects in the KB. Figure 13b shows that time grows linearly as KB grows, with the exception of early, small sizes (less than 3000 objects in the KB), where not enough instances activate the rules for two rules. The third rule, “Lack of Movement”, does not activate that much and converges to a low execution time due to not enough activations in that sample.

That led us to investigate the relation of objects in the KB to the number of objects generated, which would clearly show rule activation. Figure 13c shows more or less the same relation of linear growth for two rules which linearly generate more objects and convergence for the rule that does not.

As an interpretation, time increases due to having to generate more objects by rules activating over a larger KB. Moreover, the newly generated growing objects have to be ingested relate to Task 1.

Figure 13d–f show the same linear relation of time and objects generated. Thus, we can conclude that the major factor for time is the number of objects generated, similarly to Task 1, while the processing time for the growing KB and the actual rules firing do not significantly add to the time cost. All in all, the performance for both tasks is quite optimistic and shows that the system can scale well with increased loads of KB and rules of this type of format.

### 6.2. Clinician Focus Group Exploratory Study

Exploratory research focuses on collecting, analyzing, and interpreting observations about known designs, systems, or models, such as the framework presented in this work, with emphasis on perspective and relative importance [65]. Instead of a structured quantitative analysis, it may provide a qualitative analysis from highly specialized, small focus groups. As such, an advisory board of experts was involved in interviews to investigate preferences and advice for future clinical studies, regarding the visualization on the dashboard application and the pertaining functionality they entail in the rule-based framework. The board now includes five experts, selected for their high specialty in the field, the same advanced academic background (PhD in neuropsychology and related fields) and professional interests (care of MS and other similar neurological disorders). Lacking a standardised questionnaire for this purpose and given the explorative nature of the study, we included both closed- and open-ended questions to elicit expert advice and the level at which the framework fulfils clinician needs. As in similar studies, its results should be considered directional and descriptive, given the limited sample size [66].

The system was demonstrated to the focus group, showing a presentation of the concept, background and motivation for the system (10 min) followed by a demonstration of the two use case scenarios of Section 5.2 (5 min). Then the clinicians were free to experiment with the dashboard, answer a few questions and provide open positive and constructive feedback.

Key themes and quotes from user feedback in response to open questions are shown on Table 7 and Table 8, respectively, while the questions, average ratings and standard deviation are shown on Table 9. Overall, the reception was positive, and the interviews have shown that the clinicians saw the usefulness and the potential for professional use. Constructive comments include interesting suggestions, such as the addition of water and drug dose logging, a diary and brain sensing capabilities.

## 7. Conclusions and Future Work

In this paper, we presented a framework that utilises Semantic Web technologies to facilitate the collection, representation, aggregation and visualisation of data and information collected from wearable devices. Reusing and extending well-known ontologies and modelling patterns, interlinked KGs are generated that capture monitoring results and the semantics of the domain in a formal manner, enabling the use of SHACL rules that traverse and classify data into situations, meaningful for clinical assessment. We demonstrated the way ontologies can be used to semantically lift the domain semantics in the form of situations, combining two annotation standards (SOSA and WADM) with a rich conceptual model (DUL/DnS).

Due to the fact that the implementation is based on well-established Semantic Web technologies, it promotes interoperability, as well as fostering the use of reasoning mechanisms to identify further inferences and ensure the semantic consistency of the knowledge graphs. Furthermore, it can be easily reused and adapted to different application domains. To date, the framework has been used in MS, supporting clinical experts in easily identifying situations that might indicate problematic behaviours. Both the conceptual model and the rules can be easily configured to support the clinical assessment in other domains as well, such as dementia or in home-based rehabilitation.

Performance evaluation and an exploratory study on the usefulness perceived by clinicians have been performed using real-world data, confirming the system’s relevance in the medical practice and providing constructive feedback. In terms of performance, the system showed that it can scale linearly, with a growing number of users and objects in the KB. Regarding the real-time context, the framework has not been designed with real-time reaction scenarios in mind but rather long-term monitoring of disease progression. While sensor data arrive in near-real time (delay in the order of minutes), the inference mechanisms have to account for large periods of time (e.g., entire day or days) in order to extract health-related problems.

The achieved results are promising, but several improvements are investigated. First, the incorporation of additional sensor modalities, such as water intake, brain signals and drug doses suggested by clinicians, to enrich the collected context requires updating the underlying ontologies, both in terms of semantics and vocabulary. However, the existence of more modalities increases the complexity of the infused logic which will have a negative impact on the performance of the system. In this case, more sophisticated fusion frameworks will be required to cope with complementary and possible contradictory information. Second, the synergy with a data-driven solution is investigated, especially in cases where our knowledge-driven framework has collected and classified a large number of observations through a long monitoring period. Hybrid approaches, such as the ones presented in Section 2, have the potential to further support the clinical assessment. Due to the lack of an annotated data set (golden standard) and the deterministic nature of the proposed event-detection framework based on inference (i.e., detecting events always occurs if conditions are met), instead of validating its output, we evaluated its scalability and usefulness in an exploratory study with clinicians. As a future work, such golden standards could be obtained either from patient/carer interviews (yet time-consuming and subjective) to enable more rigorous evaluation. While this paper presents the technological framework, perspectives gathered from clinicians will be leveraged in a future clinical study to explore the system’s impact on clinical decision making and, in turn, MS care.

## Figures and Tables

**Table 1:** Data streams available from the wearable device.

Data Stream	Data Type and Metric	Resolution	Description
Steps	Number (Amount of steps)	Per Minute	An estimation of physical activity levels in “steps” per minute
Sleep Duration	Number (Time in milliseconds)	Per Sleep Session	Total sleep duration for every continuous sleep session
Minutes to Fall Asleep	Number (Time in minutes)	Per Sleep Session	Total minutes to fall asleep (awake in bed until first sleep occurrence) in a sleep session
Minutes in Deep/Light/Rem/Awake	Number (Time in minutes)	Per Sleep Session	Total minutes in sleep stage (Deep/Light/Rem/Awake) during a sleep session
Number of Awakenings	Number (Amount of awakenings)	Per Sleep Session	Number of awakenings (sleep interruptions) during a sleep session
Heart Rate	Number	Per Minute	Heart rate measurement per minute
Minutes in Fat Burn/Cardio/Peak	Number (Time in minutes)	Per Exercise Session	Total minutes in fat burn/cardio/peak heart rate zone during an exercise session

**Table 2:** Complex class descriptions (DL axioms) of basic observation types.

Class	DL Axioms
HeartObservation	$\begin{array}{c} \mathrm{HeartObservation}\equiv \mathrm{Observation}⊓\exists \mathrm{observedProperty}.\mathrm{HeartProperty}\\ \;\;\;\;⊓\exists \mathrm{hasFeatureOfInterest}.\{\mathrm{heart}\} \end{array}$
Cardio	$\begin{array}{c} \mathrm{Cardio}\equiv \mathrm{HeartObservation}⊓\exists \mathrm{observedProperty}.\{\mathrm{cardio}\} \end{array}$
FatBurn	$\begin{array}{c} \mathrm{FatBurn}\equiv \mathrm{HeartObservation}⊓\exists \mathrm{observedProperty}.\{\mathrm{fat}\_\mathrm{burn}\} \end{array}$
HeartRate	$\begin{array}{c} \mathrm{HeartRate}\equiv \mathrm{HeartObservation}⊓\exists \mathrm{observedProperty}.\{\mathrm{heart}\_\mathrm{rate}\} \end{array}$
Peak	$\begin{array}{c} \mathrm{Peak}\equiv \mathrm{HeartObservation}⊓\exists \mathrm{observedProperty}.\{\mathrm{peak}\} \end{array}$
MovementObservation	$\begin{array}{c} \mathrm{MovementObservation}\equiv \mathrm{Observation}⊓\exists \mathrm{observedProperty}.\mathrm{MovementProperty}\\ \;\;\;\;⊓\exists \mathrm{hasFeatureOfInterest}.\{\mathrm{movement}\} \end{array}$
MovementIntensity	$\begin{array}{c} \mathrm{MovementIntensity}\equiv \mathrm{MovementObservation}⊓\exists \mathrm{observedProperty}.\\ \;\;\;\;\{\mathrm{fair}\_\mathrm{activity},\mathrm{high}\_\mathrm{activity},\mathrm{light}\_\mathrm{activity},\mathrm{sedentary}\} \end{array}$
WalkingFeature	$\begin{array}{c} \mathrm{WalkingFeature}\equiv \mathrm{MovementObservation}\\ \;\;\;\;⊓\exists \mathrm{observedProperty}.\{\mathrm{distance},\mathrm{elevation},\mathrm{floors},\mathrm{steps}\} \end{array}$
SleepObservation	$\begin{array}{c} \mathrm{SleepObservation}\equiv \mathrm{Observation}⊓\exists \mathrm{observedProperty}.\mathrm{SleepProperty}\\ \;\;\;\;⊓\exists \mathrm{hasFeatureOfInterest}.\{\mathrm{sleep}\} \end{array}$
AsleepCount	$\begin{array}{c} \mathrm{AsleepCount}\equiv \mathrm{SleepObservation}⊓\exists \mathrm{observedProperty}.\{\mathrm{asleep}\_\mathrm{count}\} \end{array}$
AsleepDuration	$\begin{array}{c} \mathrm{AsleepDuration}\equiv \mathrm{SleepObservation}⊓\exists \mathrm{observedProperty}.\{\mathrm{asleep}\_\mathrm{duration}\} \end{array}$
AwakeCount	$\begin{array}{c} \mathrm{AwakeCount}\equiv \mathrm{SleepObservation}⊓\exists \mathrm{observedProperty}.\{\mathrm{awake}\_\mathrm{count}\} \end{array}$
AwakeDuration	$\begin{array}{c} \mathrm{AwakeDuration}\equiv \mathrm{SleepObservation}⊓\exists \mathrm{observedProperty}.\{\mathrm{awake}\_\mathrm{duration}\} \end{array}$

**Table 3:** Conceptual mapping of the core Descriptive Context model to the Web Annotation Vocabulary.

#DL Axiom
ms:Problem ⊑ oa:Annotation
ms:interprets ⊑ oa:hasTarget
ms:hasView ⊑ oa:hasBody

**Table 4:** A priori rule base of the different semantic rules that describe the modelled problems.

#Variables	#Rule	#Problem
Duration in seconds	Time to fall asleep in a day > 1800	Insomnia
Count of sleep interruptions	Number of interruptions in a day > 10	Restlessness
Duration in minutes	Sleep total duration in a day > 480	Too Much Sleep
Duration in minutes	Sleep total duration in a day < 300	Lack of Sleep
Duration of “Nap” state in minutes	Asleep in Naps > 100 in a day	Increased Napping
Occurrence of “Nap” State, Occurrence of “Night Sleep” state	Asleep in Naps end time < 2 h from Sleep start time	Nap close to bedtime
Time Asleep / Time in bed	Sleep Efficiency < 85	Low Sleep Quality
Step count, Heart Rate measure, Duration in minutes	Steps < 50 & Heart Rate > 90 (Fat Burn Zone) for duration > 300	Stress or Pain
Heart Rate measure	HR < 60	Low Heart Rate
Step count, Heart Rate measure, Duration in minutes	Steps < 1000 & Heart Rate < 80 for duration > 300	Inactivity
Step count, Heart Rate measure, Duration in minutes	Steps < 500 & Heart Rate < 100 for duration > 800	Lack of Movement
Step count	Steps < 8000	Lack of Exercise

**Table 5:** Time for an increasing number of objects to ingest in the KB.

No of Objects Added	Time (ms)
50	1954
100	2619
500	4548
1000	7518
3000	18,072
10,000	58,597
20,000	114,435

**Table 6:** Number of objects generated and execution time, for an increasing number of objects in the KB and for each rule.

#Objects in KB	Time (ms)	#Objects Generated
**Lack of Movement**		
50	20	0
100	77	1
500	97	1
1000	133	2
3000	301	4
10,000	519	9
20,000	1022	17
**Low Sleep Quality**		
50	8	0
100	4	0
500	7	0
1000	6	0
3000	21	0
10,000	1815	27
20,000	3047	51
**Lack of Movement**		
**Lack of Sleep**		
50	7	0
100	4	0
500	6	0
1000	61	1
3000	282	4
10,000	2500	36
20,000	4078	68

**Table 7:** Key themes and quotes from positive user feedback in response to the question: “Which needs do you think that the system currently covers for the care aspects of MS for clinicians, and how”?

Key Themes	User Quotes
Objective Monitoring	“Provides objective metrics related to sleep and physical activity for MS”, “(Covers the need) to detect physical activity, sleep and sleep quality patterns in real time.”
Prediction	“Useful for prediction”, “The system provides a basis for prediction or prevention of a possible deterioration”
Motivation	“Could help to: Address Fatigue - monitor sleep patterns and address any issues, Stay active - days with less steps and exercise could be monitored to increase stamina and strength.”
Personalization	“Identifying any contributing factors, could help clinicians to develop a tailored management plan.”

**Table 8:** Key themes and quotes from constructive user feedback in response to the question: “Which needs of clinicians do you think that the system does not cover currently and what could be done differently to improve care?”.

Key Themes	User Quotes
Add EEG for richer stress monitoring	“Brain waves (via EEG/sensors could be added) to assess stress during the day not only based on the heart rate.”
Prescription Diary Comparison	“Maybe a diary (could be added) with medical prescription (drug doses per day) in order to compare biomarkers of daily life to prescription data and predict periods of deterioration.”
Add Water Consumption to regulate Bladder/bowel function	“Issues with bladder and bowel function can be a common problem for people with MS at some stage in their life, so water consumption measurements could be added in the future to monitor this problem and give the appropriate suggestions.”

**Table 9:** Questions on a Likert-scale of 1 (Not at All) to 5 (Very Much) and user response Mean and Standard Deviation (SD).

Question	Mean	SD
How useful do you feel that the data collected by the wearable sensor (steps as a measure of physical activity, sleep, heart rate) are for monitoring & care of people with MS?	4.6	0.55
How useful do you feel that the events detected by the system (problems related to activity and sleep, e.g., lack of movement, lack of sleep, too much sleep, insomnia, restlessness, bad sleep quality) are for monitoring & care of people with MS?	4.8	0
In terms of the user interface (clinician dashboard), how easy do you think it would be for you to use in your practice?	4.4	0.55
In terms of the user interface (clinician dashboard) appearance and data representation (graphics and graphs), how appealing and easy to understand do you think it is?	4.6	0.55
Overall, how much of a positive impact do you feel that the use of the system would have in the care of MS?	4.4	0.55

## Data Availability

Not applicable.

## Abbreviations

The following abbreviations are used in this manuscript:

IoT	Internet of Things
KGs	Knowledge Graphs
MS	Multiple Sclerosis
DL	Description Logics
SOSA	Sensor, Observation, Sample, and Actuator
DUL	DOLCE+DnS Ultralite
WADM	Web Annotation Data Model
DnS	Descriptions and Situations
GUI	Graphical User Interface
OWL	Web Ontology Language
